# Augmented reality vs CAD/CAM system in orthognathic surgery: development and accuracy evaluation

**DOI:** 10.1186/s40902-025-00471-y

**Published:** 2025-10-15

**Authors:** Federica Civita, Ilaria Rota, Diego Sergio Rossi, Alberto Sinatra, Giada Anna Beltramini, Alessandro Remigio Bolzoni

**Affiliations:** 1 Maxillofacial and Dental Unit, Fondazione IRCCS Ca’ Granda Ospedale Maggiore Policlinico, Milan, Italy; 2 Department of Chemistry, Materials and Chemical Engineering “Giulio Natta” – LaBS (Laboratory of Biological Structures Mechanics), Politecnico di Milano, Milan, Italy; 3 Department of Biomedical, Surgical and Dental Sciences, University of Milan, Milan, Italy

**Keywords:** Augmented reality, Orthognathic surgery, Computer-assisted surgery, Maxillofacial surgery

## Abstract

**Background:**

Recent advancements in augmented reality (AR) have gained increasing interest in surgical applications, particularly in maxillofacial surgery. This study evaluates the accuracy and reproducibility of an AR-based navigation system using optical see-through (OST) headsets, in comparison to traditional CAD/CAM-guided approaches for Le Fort I osteotomy. Twenty identical stereolithographic skull models were divided into two groups: one group treated with OST-based AR navigation system to visualize virtual surgical lines on the skull models (*n* = 10) and the other treated with the conventional CAD/CAM procedure using titanium materialise surgical guides (*n* = 10). Virtual surgical planning (VSP) was carried out identically for both groups.

**Results:**

Surgical accuracy was assessed by measuring deviations (in mm) between planned and postoperative positions of seven cephalometric reference points. All models in the CAD group achieved deviations below the 2-mm clinical threshold (mean deviation: 0.72 ± 0.38 mm). However, only 4 out of 10 in the OST group met this threshold (mean deviation: 2.27 ± 1.24 mm). A statistical analysis using Mann–Whitney *U*-test with a significance level of 0.05 was carried out to compare the mean accuracy between OST and CAD groups. The results revealed a statistically significant difference between OST and CAD groups (*p*-value < 0.005). Nevertheless, progressive improvements in the OST group were observed, likely reflecting a learning curve associated with the new technology.

**Conclusions:**

Although CAD/CAM remains more accurate, the AR-based system offers advantages in real-time visualization and reduced costs and preoperative time by eliminating the need for 3D-printed guides. However, the current accuracy limitations highlight the need for further refinement of AR systems and increased operator training. Future studies are needed to validate the clinical applicability and reliability of AR-guided orthognathic surgery.

## Background

In recent years, augmented reality (AR)-based technologies have undergone significant development and attracted growing interest, leading to numerous studies and applications across various surgical disciplines, including maxillofacial surgery [1–5]. Currently, most orthognathic procedures are performed using computer-aided design and computer-aided manufacturing (CAD/CAM) technologies, which involve virtual surgical planning (VSP) using dedicated simulation software and the fabrication of patient-specific materialise surgical guides through three-dimensional (3D) printing. Despite their advantages, the use of physical guides may present certain limitations, such as interference with surrounding soft tissues due to their bulk. Additionally, the production process can be time-intensive and is frequently associated with increased costs [6].

An alternative solution may be represented by virtual navigation systems, which offer promising results in terms of accuracy and reliability by integrating and superimposing virtual information onto the real surgical environment. The Le Fort I osteotomy performed using optical see-through (OST) head-mounted displays follows the same steps and principles as procedures conducted with CAD/CAM systems. However, unlike the latter — which rely on physical cutting guides to perform osteotomies and drilling — the AR visor-based system projects virtual cutting lines directly onto the patient’s maxilla. These virtual lines precisely indicate the direction and length of the osteotomy, as well as the exact locations for drilling [7].

To ensure accurate alignment of the projected images with the patient’s anatomy, the system utilizes markers placed on the patient’s dental occlusion. These markers enable the headset to precisely calibrate the projection of the virtual guides, thereby ensuring an accurate overlay of the digital image onto the patient’s anatomical structures [8].

Several studies have highlighted the promising role of OST-based AR systems in orthognathic surgery [1, 8, 9].

This study aims to evaluate the effectiveness of AR-based navigation systems in achieving high accuracy and reproducibility in orthognathic surgery. In addition, it presents preliminary comparative data between OST-based AR system and conventional CAD/CAM approach, focusing on surgical precision and procedural efficiency.

## Methods

For the present study, 20 identical dry-bone skull models (provided by SYNBONE AG Tardisstrasse 199 7205 Zizers, Switzerland), replicating the anatomy of an adult patient with a full permanent dentition, were used. All models were radiopaque and with an adequate resistance to cutting with a piezoelectric surgical instrument, ensuring their suitability for orthognathic surgical procedure simulation. A total of 10 identical Materialise surgical guides and 10 identical occlusal splints, each equipped with 6 spherical markers for AR navigation, 20 identical Materialise titanium 3D printed personalised plates specifically designed for a Le Fort I osteotomy, and a wearable visor featuring optical see-through technology for AR-based surgical guidance (Microsoft HoloLens 2), were employed in this work.

All materials were supplied by Corios Network.

The 20 stereolithographic models were divided into two experimental groups:

- *OST group (n* = *10)*: These models underwent surgery using virtual materialise surgical guides. The surgical plan was uploaded to OST augmented reality visors, enabling intraoperative guidance without the use of physical materialise surgical guides. Osteotomies and drill holes for the placement of the Materialise titanium 3D printed personalised plates were performed under AR guidance, following alignment of the virtual surgical plan (VSP) with the anatomical model.
- *CAD group (n* = *10)*: These models underwent the standard CAD/CAM procedures using physical Materialise titanium 3D printed personalised surgical guides. These were positioned and fixed onto the models to guide the osteotomies and screw holes for the Materialise titanium 3D printed personalised plates.

To perform the surgical planning, a skull model underwent a CT scan at Sirio s.r.l. in Milan with 0.4 mm of pixel spacing and slice thickness, followed by segmentation using Mimics InPrint 3.0 (Materialise®, Leuven, Belgium) and generation of the corresponding STL file.

### Virtual surgical planning

**Fig. 1:**
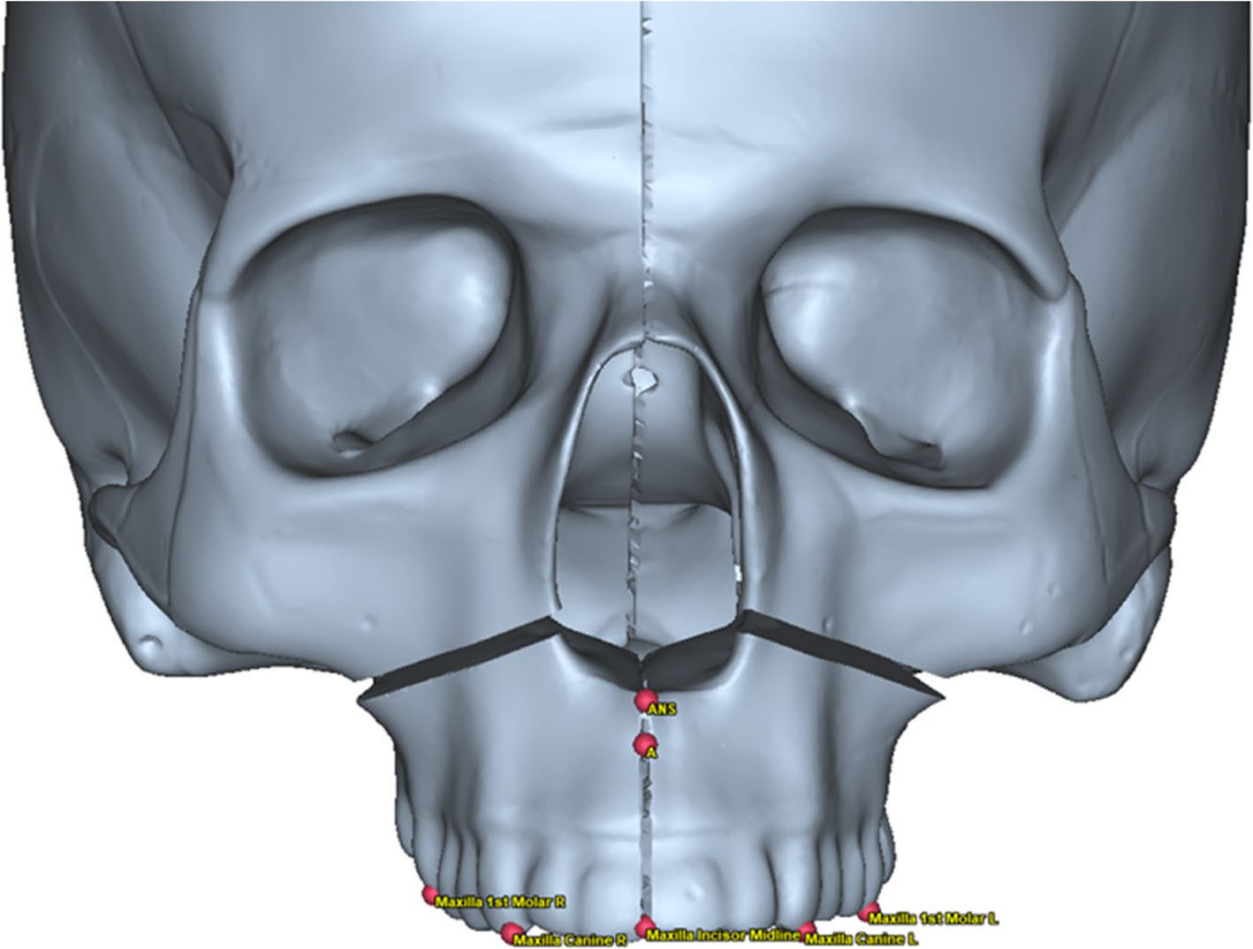
Seven cephalometric reference points on the maxilla and the maxillary dental arch (red spots)

The virtual surgical planning of Le Fort I was carried out, using PROPLAN CMF 3.0.1 (Materialise, Leuven, Belgium). Seven cephalometric reference points, which are not involved in the surgical procedure, were identified on the maxilla and the upper dental arch. As shown in Fig. 1, the points are as follows:

- The deepest point on the curvature of the maxillary alveolar process incisors (PA)
- Mesiobuccal cusp of right maxillary first molar
- Mesiobuccal cusp of left maxillary first molar
- Superior median interincisor line
- Cusp of right maxillary canine
- Cusp of left maxillary canine
- The extreme anterior point on the maxilla called anterior nasal spine (ANS)

The planned movements of the cephalometric points in the three directions are reported in Table 1.

**Table 1:** Virtual surgical planned maxillary movements

Point	*X* (mm)	*Y* (mm)	*Z* (mm)
PA	0	3 anterior	3 down
Right maxillary first molar	0	3 anterior	3 down
Left maxillary first molar	0	3 anterior	3 down
Superior median interincisor line	0	3 anterior	3 down
Right maxillary canine	0	3 anterior	3 down
Left maxillary canine	0	3 anterior	3 down
ANS	0	3 anterior	3 down

### Surgical procedure on skull models

**Fig. 2:**
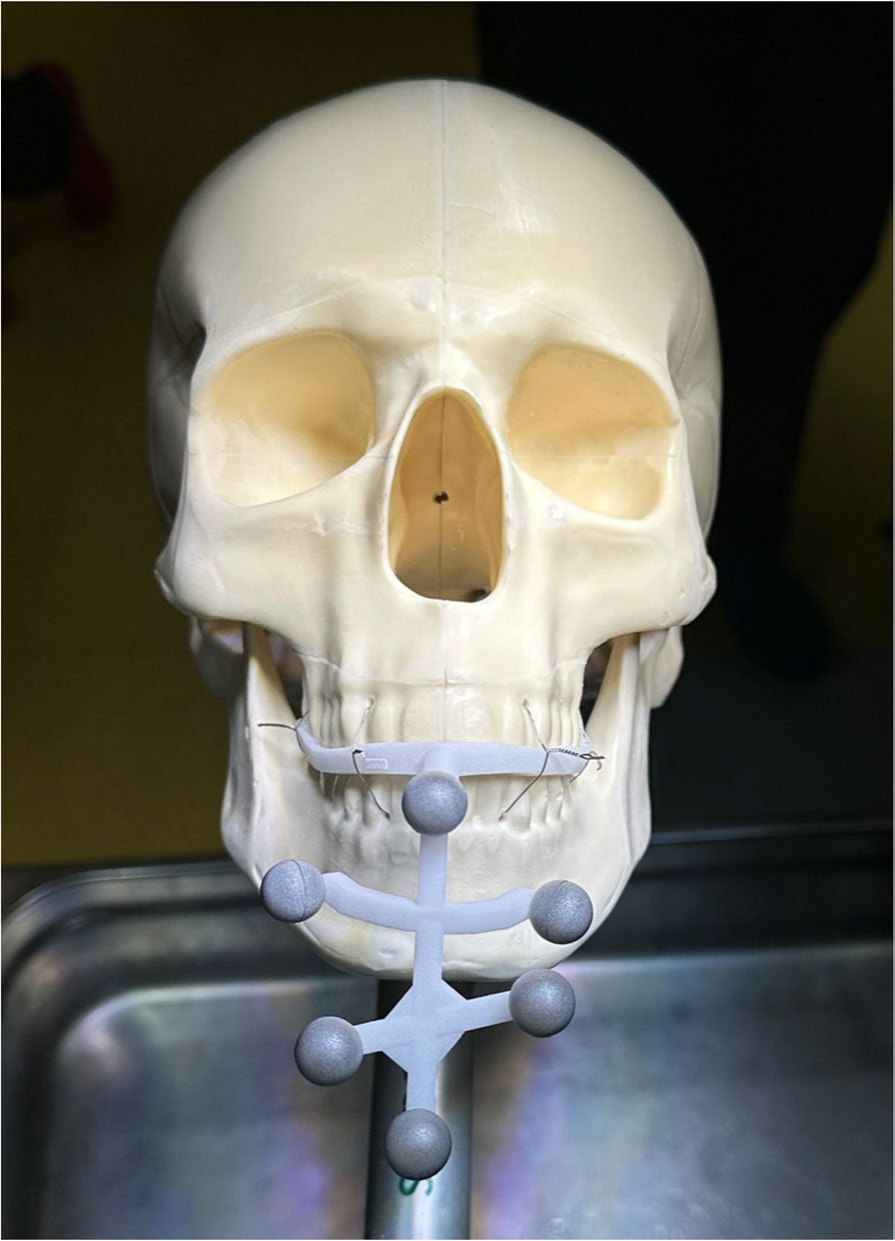
Skull model of the OST group equipped with an occlusal splint, firmly secured to the maxillary dental arch using metallic wires

The OST skull models were equipped with occlusal splints, which were securely anchored to the dental arches using metallic wires, as illustrated in Fig. 2. The experimental environment was prepared within an operating room to closely simulate clinical conditions. Each skull model was placed on the surgical table in a position replicating the typical orientation of a patient during surgery, allowing the surgeon to operate from a realistic point of view.

Prior to initiating the surgical procedure on the OST models, the HoloLens visor was properly calibrated and adjusted on the surgeon’s head to ensure optimal alignment and comfort.

All procedures (both in the OST and CAD groups) were performed by the same expert maxillofacial surgeon. A standard Le Fort I osteotomy was executed in accordance with the preoperative virtual surgical planning to maintain consistency across all models and to allow for comparative evaluation between the two groups.

### Accuracy evaluation

Postoperative CT scans were performed at Sirio s.r.l in Milan on each model, and the bone segmentation was carried out in Mimics inPrint to isolate bone segments from patient-specific titanium plates and fixation screws. Then, postoperative anatomies were aligned to virtual planned skull model using PROPLAN CMF 3.0.1 surface automatic alignment tool.

For each model in both groups, deviations (in millimeters) between the planned reference points in the VSP and the corresponding points in the postoperative models were recorded.

**Fig. 3:**
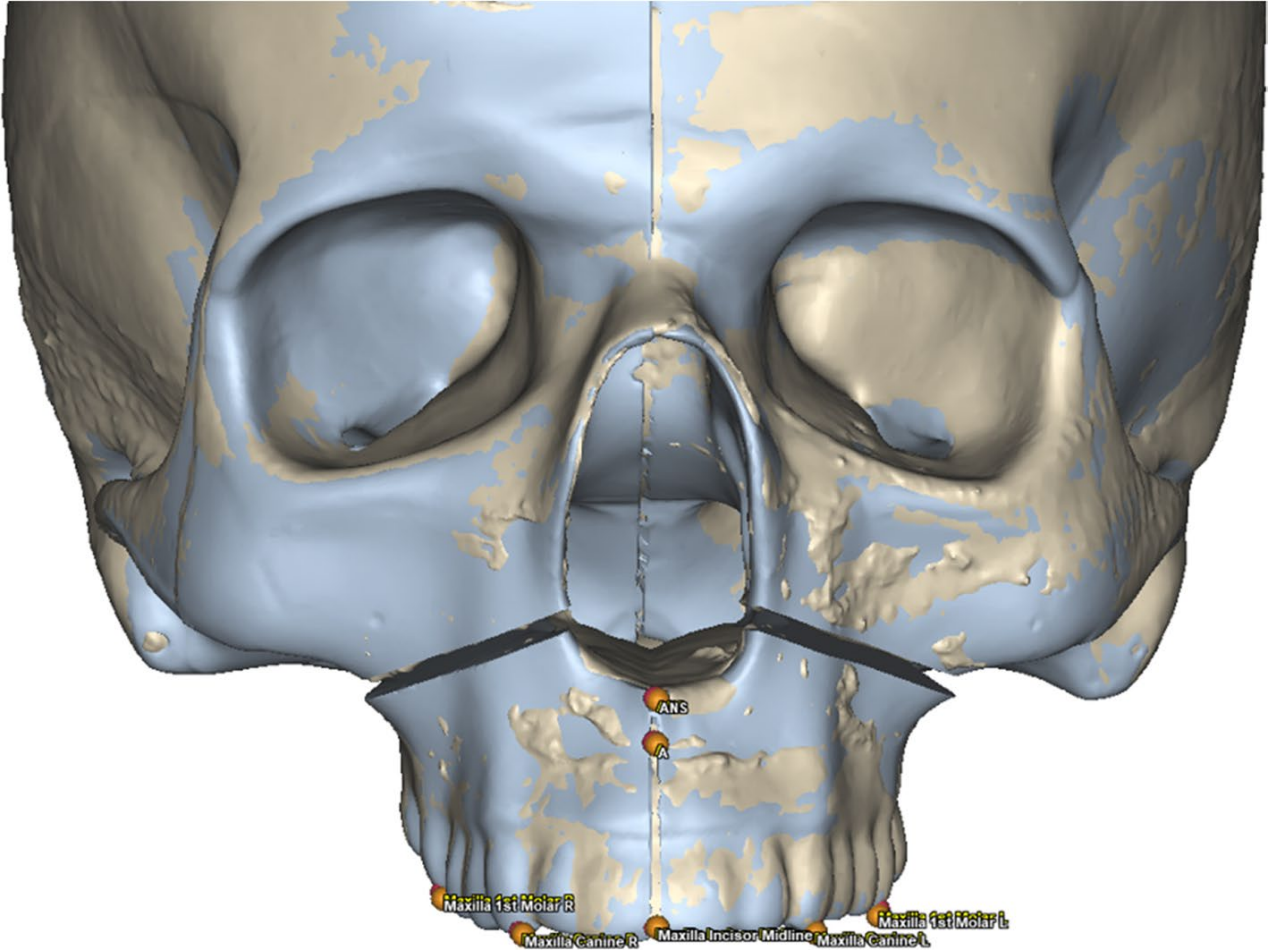
Alignment between postoperative skull model and virtual planned skull model. Red spots indicate the virtual position on the reference points after the VSP, and orange spots indicate the real postoperative position of the reference points

Figure 3 shows an example of alignment between the virtual surgical planning and a postoperative skull model.

## Results

To evaluate the accuracy of Le Fort I surgery, the point-wise distance of the seven reference points between virtual planned and postoperative anatomies was measured.

The results of the accuracy evaluation in OST and CAD groups, as differences between virtual planning and real outcomes for each point, are reported in Tables 2 and 3, respectively.

**Table 2:** Point-wise deviations and overall mean difference between virtual planning and postoperative outcomes in the OST group

Model	PA (mm)	Right maxillary first molar (mm)	Left maxillary first molar (mm)	Superior median interincisor line (mm)	Right maxillary canine (mm)	Left maxillary canine (mm)	ANS (mm)	Mean deviation ± standard deviation (mm)
MOD01OST	0.58	1.30	1.48	0.86	1.07	1.09	0.51	0.98 ± 0.36
MOD02OST	4.40	9.47	4.04	4.30	7.01	1.23	4.40	4.98 ± 2.59
MOD03OST	1.61	2.45	2.30	2.52	2.68	2.24	1.43	2.18 ± 0.47
MOD04OST	1.68	3.04	1.81	2.38	2.73	1.91	1.57	2.16 ± 0.56
MOD05OST	2.68	5.11	1.75	3.62	4.63	1.84	2.53	3.17 ± 1.32
MOD13OST	1.23	1.23	2.56	1.94	2.29	2.18	1.11	1.97 ± 0.58
MOD16OST	1.21	1.21	1.32	1.52	2.02	0.95	1.24	1.49 ± 0.45
MOD17OST	1.41	1.41	2.69	2.51	2.62	2.37	1.24	2.20 ± 0.61
MOD18OST	2.31	2.31	2.78	3.44	3.87	2.31	2.37	2.94 ± 0.65
MOD19OST	0.42	0.42	0.86	0.55	0.61	0.66	0.40	0.60 ± 0.16

**Table 3:** Point-wise deviations and overall mean difference between virtual planning and real outcomes in the CAD group

Model	PA (mm)	Right maxillary first molar (mm)	Left maxillary first molar (mm)	Superior median interincisor line (mm)	Right maxillary canine (mm)	Left maxillary canine (mm)	ANS (mm)	Mean deviation ± standard deviation (mm)
MOD06CAD	0.34	0.47	0.71	0.53	0.42	0.72	0.35	0.51 ± 0.16
MOD07CAD	0.27	0.54	0.31	0.45	0.50	0.34	0.23	0.38 ± 0.12
MOD08CAD	0.22	0.48	0.73	0.49	0.43	0.59	0.17	0.44 ± 0.20
MOD09CAD	0.70	0.97	0.86	1.11	1.13	0.94	0.64	0.91 ± 0.19
MOD10CAD	0.90	1.72	1.73	1.64	1.81	1.52	0.74	1.44 ± 0.43
MOD11CAD	0.59	1.24	0.92	0.92	1.08	0.82	0.52	0.87 ± 0.25
MOD12CAD	0.40	0.19	0.46	0.41	0.30	0.46	0.41	0.38 ± 0.10
MOD14CAD	0.32	0.73	0.65	0.23	0.26	0.39	0.42	0.43 ± 0.19
MOD15CAD	0.72	1.58	1.63	1.32	1.42	1.39	0.60	1.24 ± 0.41
MOD20CAD	0.75	0.91	0.57	0.80	0.65	0.41	0.50	0.66 ± 0.18

The 70 individual measurements and the 10 mean deviations calculated for each model in OST and CAD groups have been compared to the reference threshold of 2 mm reported in the literature for different maxillofacial surgeries [5, 8, 10–14].

A total mean accuracy was calculated as the average of the mean deviation for all models in a group. The total mean accuracies in the OST and CAD groups are 2.27 mm ± 1.24 mm and 0.72 mm ± 0.38 mm, respectively. A statistical analysis using Mann–Whitney *U*-test with a significance level of 0.05 was carried out to compare the mean accuracy between OST and CAD groups. The analysis revealed a statistically significant difference between OST and CAD groups (*p*-value < 0.005) highlighting that the accuracy of the CAD group is higher than that of the OST group.

For a more detailed analysis, the mean differences of the seven reference points were compared between the OST and CAD groups. Mann–Whitney *U*-test (significance level equal to 0.05) was applied to compare the mean deviations between OST and CAD groups for each of the seven reference points.

The results are summarized in Table 4 which shows the mean differences and standard deviations for each reference point in both groups. In general, the CAD group demonstrated significantly lower deviations compared to the OST group for all reference points.

**Table 4:** Mean deviation and standard deviation of the seven reference points in the OST and CAD groups

Group	PA (mm)	Right maxillary first molar (mm)	Left maxillary first molar (mm)	Superior median interincisor line (mm)	Right maxillary canine (mm)	Left maxillary canine (mm)	ANS (mm)
OST	1.75 ± 1.16	3.28 ± 2.48	2.16 ± 0.91	2.36 ± 1.20	2.95 ± 1.85	1.68 ± 0.64	1.68 ± 1.17
CAD	0.50 ± 0.23	0.87 ± 0.50	0.89 ± 0.46	0.77 ± 0.46	0.82 ± 0.53	0.78 ± 0.40	0.45 ± 0.18
*p*-value	0.0011	0.0007	0.0019	0.0015	0.0011	0.0021	0.0036

## Discussion

Augmented reality is gaining increasing interest in the surgical field in recent years.

CAD/CAM technologies — including virtual surgical planning (VSP) and the production of patient-specific 3D-printed guides — have become standard in clinical practice due to their high degree of accuracy and reliability, as confirmed by numerous studies [15, 16]. These systems allow for precise osteotomies and improved reproducibility, particularly in Le Fort I procedures. However, their dependence on physical guides, which may interfere with soft tissues, and the time and cost burden associated with guide fabrication, has led to increased interest in digital solutions that can streamline the workflow without compromising precision [7].

In this context, AR-based navigation systems are presented as a forward-looking solution. Although still in early clinical stages, they offer several potential advantages: real-time adaptability, elimination of physical guide-related obstructions in the surgical area, and a reduction in preoperative preparation time.

AR integrates virtual information into the real environment, allowing the overlay of a virtual anatomical model onto the physical one. This enables surgeons to visualize osteotomy lines and screw hole positions directly on the real anatomy, eliminating the need for physical materialise surgical guides. Moreover, thanks to the transparent lenses of AR visors, the surgeon maintains their natural line of sight. Furthermore, during surgical navigation, AR helps avoid the constant need for the surgeon to shift their attention between the patient and a separate screen.

The literature highlights the potential of augmented reality in maxillofacial surgery, with numerous studies reporting encouraging accuracy outcomes under controlled conditions. For example, Ayoub et al. (2019) demonstrated sub-millimetric deviations in osteotomy execution using AR-guided navigation on cadaveric models, indicating that AR technology may approach the precision levels achieved by conventional methods [1]. Likewise, Pietruski et al. (2019) confirmed the feasibility and accuracy of AR-assisted procedures employing OST head-mounted displays, illustrating that surgeons could effectively follow virtual planning without the aid of physical guides [17]. Moreover, Ruggiero et al. (2023) reported promising accuracy results of an in vitro study for the use of augmented reality in pediatric craniofacial surgery [5].

This study introduces several aspects that differentiate it from previous research on augmented reality (AR) in maxillofacial surgery. Moreover, this work provides a direct and quantitative comparison between the AR navigation system with OST headsets and the traditional CAD/CAM approach, using identical anatomical models. This offers a unique and precise view of the accuracy differences between the two technologies.

In this regard, notable differences were found in surgical accuracy between the OST and CAD groups when compared to the virtual surgical planning. Within the OST group, only 4 out of 10 models demonstrated a deviation < 2 mm from the VSP. This threshold of 2 mm is commonly regarded in the cranio-maxillofacial surgical literature as the upper limit for clinically acceptable accuracy and thus is used as a benchmark for surgical success [5, 8, 10–14]. The remaining six models in the OST group exceeded this threshold. Specifically, in three of these six cases, the deviation ranged between 2 mm and 2.5 mm, while in the other three models the deviation was > 2.5 mm. Such discrepancies suggest suboptimal surgical outcomes and raise concerns about the reliability of the OST approach in achieving precise Le Fort I osteotomy.

One particularly notable outlier was the MOD02OST model, which showed the highest deviations from the VSP among all OST-treated cases. The deviation observed in this model was significantly greater than that of any other in the OST group. This anomaly is likely attributable to a misalignment between the physical and virtual models during the registration phase (the superposition between real and virtual skull), which was not corrected prior to the execution of the Le Fort I osteotomy. This highlights the critical importance of ensuring accurate initial registration between real and virtual anatomy when using AR-based guidance, as early inaccuracies can propagate throughout the procedure and substantially compromise the outcome. Additionally, among the first five OST models treated, only one achieved a deviation of less than 2 mm, indicating that acceptable surgical accuracy was initially difficult to obtain. In contrast, within the latter five cases, three models fell within the clinically acceptable range. This improvement over time may reflect increasing operator familiarity with the use of AR visors technique and workflow, suggesting that surgical proficiency and experience can significantly influence the outcomes of maxillofacial Le Fort I surgery. However, even with this apparent improvement, the overall reliability of OST remains questionable when compared to computer-aided methods.

In contrast, the CAD group demonstrated significantly higher accuracy and consistency. Among the CAD models, 8 out of 10 exhibited deviations from the VSP of less than 1 mm, indicating a high level of surgical precision. The remaining two models presented deviations between 1 and 1.5 mm, which still fall within an acceptable range according to established criteria. Moreover, it is worth noting that all measured points across the CAD models exhibited a difference from the *VSP* < 2 mm. These results of accuracy evaluation are in accordance with results reported in the literature and support its clinical reliability in maxillofacial surgical applications using CAM/CAD technologies [6, 10–12, 14, 18–21].

## Conclusion

Although the OST system showed some limitations in terms of surgical accuracy, the overall results still encourage optimism for the future of OST technology in maxillofacial surgery. The improvement observed in later models highlights the role of operator experience and suggests that with further refinement and training, OST-guided surgery has the potential to reach accuracy levels comparable to CAD/CAM techniques.

Moreover, despite the observed deviations, the OST approach offers certain unique advantages that should not be overlooked. The potential for real-time visualization, reduction of physical hardware in the operating field, and adaptability to different surgical situations remain compelling reasons to continue exploring and refining AR-assisted techniques. Furthermore, as AR technology continues to evolve, it is possible that future advancements in both hardware and software could address the current limitations, improving accuracy and reproducibility. Notably, the OST approach also offers a significant cost and time-saving advantage, as it eliminates the need for the design and the 3D printing of custom materialise surgical guides, reducing material costs and streamlining the preoperative process.

However, in contrast to CAD/CAM solutions — whose clinical efficacy has been substantiated by extensive in vivo research and long-term follow-up — AR technologies still lack robust, large-scale clinical trials necessary to confirm their consistency, usability, and surgical benefit in routine practice.

In conclusion, although CAD/CAM systems continue to outperform in terms of accuracy and reliability, OST-based navigation presents a promising alternative due to its minimally invasive nature and greater intraoperative flexibility. As the technology evolves and surgeons gain experience with its application, OST-guided procedures are likely to become a more practical option, especially in cases where reduced setup time, cost-effectiveness, and adaptability are essential. Future directions should focus on training for surgeons and facilitating the transfer of AR technologies into real-world clinical settings. For this reason, further studies are needed to optimize the technique and assess its effectiveness across a wider range of clinical scenarios ensuring that OST systems reach their full potential in surgical practice.

## Data Availability

The datasets used and/or analysed during the current study are available from the corresponding author on reasonable request.

## Abbreviations

3D: Three-dimensional
ANS: Anterior nasal spine
AR: Augmented reality
CAD: Computer-aided design
CAM: Computer-aided manufacturing
CT: Computed tomography
OST: Optical see-through
PA: Point A (deepest point on the curvature of the maxillary alveolar process incisors)
